# Les 10 ans du Fonds Français Muskoka. Bilan et perspectives

**DOI:** 10.48327/mtsi.v2i4.2022.286

**Published:** 2022-11-17

**Authors:** Fleur VERNAT

**Affiliations:** Coordinatrice du Secretariat du Fonds Français Muskoka

**Keywords:** Muskoka, SRMNIA-Nut (Santé reproductive, maternelle, néonatale, infantile, des adolescents et de nutrition), Partenariat, IHI (Interventions à haut impact), Gouvernement français, Muskoka, SRMNIA-Nut (Reproductive, maternal, newborn, child, adolescent and nutrition health), Partnership, IHI (high impact interventions), French government

## Abstract

Créé lors du G8 de 2010, le Fonds Français Muskoka représente l'engagement de la France en faveur de la santé maternelle et infantile. Ce mécanisme de coordination innovant entre quatre agences des Nations unies (OMS, ONU Femmes, UNICEF et UNFPA) travaille depuis 10 ans à l'amélioration de la santé et du bien-être des mères, des nouveaunés, des enfants, des adolescents et des jeunes dans 9 pays d'Afrique de l'Ouest et du Centre: Bénin, Burkina Faso, Côte d'Ivoire, Guinée, Mali, Niger, Sénégal, Tchad et Togo. Cette rare longévité s'explique notamment par une structure de gouvernance innovante, une grande capacité d'adaptation au contexte local, un effet de levier au travers d'interventions à haut impact ainsi que ses résultats sur le terrain. Alors que le Fonds Français Muskoka a été renouvelé pour 5 nouvelles années par la France en 2021, un processus de réflexion a été lancé afin d'accroître l'effet catalytique de cette initiative efficace et prometteuse.

## Abstract (en)

Created at the 2010 G8, the French Muskoka Fund represents France's commitment to maternal and child health. This innovative coordination mechanism between four UN agencies (WHO, UN-Women, UNICEF and UNFPA) has helped improving the health and well-being of mothers, newborns, children, teenagers and young people in 9 countries of Western and Central Africa (Benin, Burkina Faso, Chad, Côte d'Ivoire, Guinea, Mali, Niger, Senegal, and Togo) for the past 11 years. This rare longevity can be explained by its innovative governance structure, its ability to adapt to the local context, its capacity to leverage through high-impact interventions, and its impact on the ground. As the French Muskoka Fund has been renewed for another five years by France in 2021, reflection has been launched to further enhance the catalytic effect of this effective and promising initiative.

## Introduction

L'Afrique de l'Ouest et du Centre concentre à elle seule 30% des cas de malnutrition sévère, 42% des décès maternels et 35% des décès chez les enfants de moins de 5 ans recensés à l’échelle mondiale.

C'est pour répondre à l'urgence de cette situation que le Fonds Français Muskoka [4] a été créé par la France lors du Sommet du G8 qui s'est tenu en 2010 à Muskoka au Canada [3]. Rassemblant 4 agences des Nations unies (OMS, ONU Femmes, UNFPA et UNICEF), cette initiative a pour objectif de renforcer les systèmes de santé pour améliorer la santé et le bien-être des mères, des nouveau-nés, des enfants, des adolescents et des jeunes et ainsi accélérer l'atteinte des Objectifs de développement durable 2, 3, 5, 10 et 17 dans 9 pays de la sous-région: Bénin, Burkina Faso, Côte d'Ivoire, Guinée, Mali, Niger, Sénégal, Tchad et Togo.

Au travers d’« Interventions à Haut Impact » (IHI, c'est-à-dire d'interventions simples et peu coûteuses mais à l'efficacité scientifiquement prouvée), le Fonds Français Muskoka intervient dans 4 domaines:

- RSS: renforcement des systèmes de santé, y compris les ressources humaines et médicaments essentiels;
- SMNI/PF: santé maternelle, néonatale et infantile et planification familiale;
- SSRAJ: santé sexuelle et reproductive des adolescents et des jeunes;
- NUT: nutrition.

Il est à noter que ces deux derniers domaines (SSRAJ et NUT) ont récemment bénéficié d'une hausse de leurs budgets – évolution qui témoigne très clairement de la priorité croissante accordée par la France à la jeunesse.

## Gouvernance

La gouvernance du Fonds Français Muskoka s'articule autour de 4 organes:

- C'est au niveau de chaque pays tout d'abord que s'effectue la mise en œuvre des programmes de renforcement des systèmes de santé. Ces activités sont coordonnées par un groupe interagences rassemblant non seulement les représentants et techniciens des 4 agences impliquées, mais également les techniciens des ministères concernés. Ce groupe se réunit régulièrement afin d'assurer la mise en œuvre la plus efficace possible sur le terrain, en parfait alignement avec les priorités nationales.
- Afin d'appuyer au mieux les équipes pays, le niveau régional mène des travaux de recherche, d'innovation et d'identification de bonnes pratiques. Ces activités régionales sont coordonnées par un Comité technique (Cotech) qui réunit des techniciens des 4 agences des Nations unies et du ministère français de l'Europe et des Affaires étrangères (MEAE), des Conseillers régionaux en santé mondiale, le Secrétariat du Fonds Français Muskoka ainsi que des représentants de la jeunesse et de la société civile.
- C'est ensuite au niveau des bailleurs – et plus précisément au sein du Comité de pilotage (Copil) – que les décisions stratégiques sont prises. Ce dernier rassemble de hauts représentants du MEAE et des pays bénéficiaires (niveau ministériel), les directeurs régionaux des 4 agences ainsi que les représentants d'autres partenaires techniques et financiers tels que l'Agence française de développement (AFD).
- Ces 3 niveaux sont coordonnés par un secrétariat qui assure le lien entre les 3 organes de gouvernance, les niveaux pays et régional ainsi qu'entre les 4 agences. Plus précisément, le secrétariat est en charge de la gestion, de la coordination, du suivi et de l’évaluation, du plaidoyer et de la visibilité du Fonds Français Muskoka.

Autre particularité, le Fonds Français Muskoka est un mécanisme de coordination novateur permettant de maximiser l'expertise technique et l'usage des ressources des 4 agences des Nations unies, tout en évitant les duplications, pour un maximum d'impact (en parfait alignement en cela avec la réforme du système des Nations unies lancée par son Secrétaire général en 2017 [8]. Ce mécanisme permet par ailleurs un suivi et un rapportage conjoint des résultats et de l'exécution financière ainsi qu'une synchronisation des activités pays et interpays.

## Programmation Et Actions

Sur le plan programmatique, les programmes du Fonds Français Muskoka sont en complet alignement avec les plans nationaux et les besoins des populations de chacun des 9 pays bénéficiaires (grâce en particulier à l’étroite collaboration précédemment citée entre techniciens des ministères et des agences au niveau des équipes pays) et reposent sur une planification intégrée et multisectorielle (en complète adéquation en cela avec l'Agenda 2030 pour le développement durable des Nations Unies [1] et l'Agenda 2063 de l'Union africaine [9]). Il existe enfin une complète synergie entre la mise en œuvre au niveau pays et le travail de recherche et d'innovation au niveau régional – ce qui permet aux programmes du FFM de constamment se renouveler en fonction de l’évolution des besoins sur le terrain et des dernières avancées scientifiques.

Ce sont ces particularités qui ont permis au Fonds Français Muskoka de perdurer 12 ans, ce qui constitue une longévité suffisamment rare pour être soulignée, en particulier pour un dispositif rassemblant autant d'intervenants dans un contexte difficile et marqué par une instabilité chronique, tant sur les plans politique, sécuritaire et institutionnel qu’économique, social et environnemental.

Tout au long de ses 12 premières années d'existence, le Fonds Français Muskoka a bien sûr eu à relever un certain nombre de défis. En interne tout d'abord, la planification annuelle et le retard des versements ont longtemps posé le défi de la rapidité du décaissement des fonds. Les cycles budgétaires des différents partenaires impliqués (France, pays partenaires, agences onusiennes et partenaires de mise en œuvre) étant rarement alignés, des goulots d’étranglement administratifs se créant aussi dans les procédures de décaissement. En termes de défis externes, l'instabilité des environnements politique, sécuritaire, économique, social, climatique et sanitaire représentent autant de freins à la mise en œuvre, de même que le poids des déterminants socioculturels, l’évolution du contexte démographique ainsi que la multiplication des partenaires techniques et financiers qui pose de façon accrue le défi de la coordination. Dans la plupart des pays bénéficiaires, la mise en œuvre des activités et le suivi des interventions se heurtent par ailleurs souvent à un manque de ressources humaines en santé, à des systèmes d'information sanitaire insuffisants, à l'irrégularité de l'approvisionnement en produits de santé et aux faibles budgets alloués par les gouvernements au secteur de la santé.

## Un Bilan Très Positif

En dépit de tous ces défis, le bilan du Fonds Français Muskoka au cours de ces 10 premières années reste cependant très largement positif, il a notamment permis [5]:

- d'harmoniser l'appui technique aux pays en matière de Santé reproductive, maternelle, néonatale, infantile et des adolescents et de nutrition (SRMNIA-Nut);
- d’élaborer conjointement des stratégies régionales et nationales complémentaires, basées sur l'expertise technique des 4 agences et reflétant les dernières directives mondiales et les connaissances scientifiques;
- d'exercer un effet catalytique sur les politiques nationales ainsi que les ressources techniques et financières;
- d'améliorer la redevabilité et la traçabilité des financements à tous les niveaux, la documentation des bonnes pratiques et la facilitation de la collaboration Sud-Sud;

## Impact Et Avenir

Au-delà de ces chiffres, il n'y a sans doute pas de meilleur indicateur de l'impact du Fonds Français Muskoka que les témoignages de nos bénéficiaires. Ainsi ce témoignage de Roubatou T., une jeune fille togolaise de 15 ans qui a découvert ses droits en fréquentant le Centre des jeunes de son quartier (soutenu par le Fonds Français Muskoka) et qui a aussi pu trouver les mots pour convaincre son père de ne pas la marier avant qu'elle ait pu finir sa scolarité – comme cela avait été le cas pour ses quatre sœurs avant elle.

Alors que nous sommes maintenant entrés dans la dernière décennie des Objectifs de développement durable et face aux nombreuses crises qui captent l'attention de la communauté internationale (comme récemment le conflit en Ukraine), le Fonds Français Muskoka incarne l'espoir en maintenant la santé maternelle et infantile au cœur de l'agenda santé des gouvernements des pays bénéficiaires et de leurs partenaires. Suite à l'annonce de son renouvellement pour 5 années supplémentaires en juillet 2021, toutes les équipes du Fonds Français Muskoka se sont lancées dans l’élaboration d'une stratégie Muskoka 3.0, et ce afin de maximiser le potentiel de cette initiative française à la fois efficace et prometteuse – si possible au travers de davantage de financements bien sûr (en particulier suite à la réintroduction du Burkina Faso et face à l'intérêt croissant suscité par ce programme), mais également au travers d'une possible ouverture a d'autre partenaires et/ou bailleurs. Toujours en termes de perspectives et opportunités, le Fonds Français Muskoka pourrait aussi davantage contribuer à une meilleure coordination de toutes les initiatives et efforts entrepris en faveur de la SRMNIA-Nut dans la région et au-delà, renforçant ainsi plus encore l'effet catalytique de cette initiative passionnante, efficace et prometteuse.

- et d'accroître l'appropriation de la SRMNIA-Nut par les décideurs politiques comme une thématique clé de santé publique.
  Le Fonds Français Muskoka a par ailleurs eu un impact certain sur les thématiques liées à la jeunesse et les questions de genre. Rappelons à ce sujet que Muskoka a été spécifiquement identifié comme l'outil de mise en œuvre de la grande cause du quinquennat du Président Macron qu'est l’Égalité entre les femmes et les hommes [2]. Enfin le Fonds Français Muskoka a su s'adapter pour maintenir ses activités et soutenir les populations tout au long des différentes crises qu'a connues la sous-région (la crise de la COVID-19 bien sûr, mais également l’épidémie d'Ebola ou encore les crises humanitaires au Mali, au Niger et au Tchad au cours de ces dernières années). Dans ce contexte difficile donc, le Fonds Français Muskoka a également permis d'atteindre des résultats concrets sur le terrain. En voici quelques exemples chiffrés:
- une réduction du taux de mortalité maternelle de 17% dans les pays bénéficiaires entre 2010 et 2017 [5, 6, 7];
- un recul de la mortalité néonatale de 22% entre 2011 et 2018 [5, 6, 7];
- la formation de plus de 70 000 personnels de santé (médecins, infirmières et infirmiers, sages-femmes et agents de santé communautaires) en 10 ans d'intervention [5];
- le quasi-doublement du taux d'accouchement assisté par un personnel de santé qualifié entre 2016 et 2020 dans les districts d'intervention du Fonds Français Muskoka au Mali [5];
- une augmentation de 68% du taux d'allaitement maternel exclusif pour les nourrissons de moins de 6 mois au Burkina Faso entre 2012 et 2020 pour atteindre 64% des nouveau-nés [5];
- ou encore la diffusion et la supervision de cours d’éducation complète à la sexualité auprès de 71,4% des nouveaux élèves du secondaire en Côte d'Ivoire [6].

## Liens D'intérêts

L'autrice ne déclare aucun lien d'intérêt.
